# Profiling the genome-wide DNA methylation pattern of porcine ovaries using reduced representation bisulfite sequencing

**DOI:** 10.1038/srep22138

**Published:** 2016-02-25

**Authors:** Xiao-Long Yuan, Ning Gao, Yan Xing, Hai-Bin Zhang, Ai-Ling Zhang, Jing Liu, Jin-Long He, Yuan Xu, Wen-Mian Lin, Zan-Mou Chen, Hao Zhang, Zhe Zhang, Jia-Qi Li

**Affiliations:** 1 Guangdong Provincial Key Lab of Agro-Animal Genomics and Molecular Breeding, National Engineering Research Centre for Breeding Swine Industry, College of Animal Science, South China Agricultural University, Guangzhou, Guangdong, China; 2 College of Biological and Food Engineering, Guangdong University of Education, Guangzhou, Guangdong, China; 3 Guangzhou Ribobio Co., Ltd., Guangzhou, Guangdong, China

## Abstract

Substantial evidence has shown that DNA methylation regulates the initiation of ovarian and sexual maturation. Here, we investigated the genome-wide profile of DNA methylation in porcine ovaries at single-base resolution using reduced representation bisulfite sequencing. The biological variation was minimal among the three ovarian replicates. We found hypermethylation frequently occurred in regions with low gene abundance, while hypomethylation in regions with high gene abundance. The DNA methylation around transcriptional start sites was negatively correlated with their own CpG content. Additionally, the methylation level in the bodies of genes was higher than that in their 5′ and 3′ flanking regions. The DNA methylation pattern of the low CpG content promoter genes differed obviously from that of the high CpG content promoter genes. The DNA methylation level of the porcine ovary was higher than that of the porcine intestine. Analyses of the genome-wide DNA methylation in porcine ovaries would advance the knowledge and understanding of the porcine ovarian methylome.

DNA methylation plays a critical function in many biological processes. It regulates gene expression, genomic imprinting, cell differentiation and embryogenesis^1^,^2^,^3^,^4^. In recent years, next-generation DNA sequencing has achieved landmark advances which have enabled the investigation of the DNA methylation dynamics of vital biological functions over time^5^,^6^.

As a biological model, pigs share many analogous developmental processes and genomic characteristics with humans^7^,^8^. Long-term selection and adaption towards high prolificacy and meat production have transformed porcine epigenetics^8^, along with associated genotypic and phenotypic changes^7^,^9^ resulting from the modification of the epigenetic regulation of chromatin structure and transcriptional activity. During the transformation process, the porcine DNA methylome displays variable patterns in different breeds and sexes of pigs as well as variation in different anatomic tissues^8^. In female mammals, ovaries determine the capacity for fertilization and the reproductive lifetime. During the process of sexual and ovarian maturation, the biological structures and follicular morphology of the ovaries dramatically transform in pigs^10^ and other mammals^11^. DNA methylation regulates the initiation of sexual and ovarian maturation in female mammals^12^,^13^. Genome-wide studies have shown that differences in DNA methylation between polycystic ovaries and normal ovaries are associated with genes involved in multiple signaling pathways that are crucial for ovarian maturation, including the p53 and the NOD-like receptor signaling pathways^14^,^15^. These studies have improved our understanding of the basic epigenetic molecular mechanisms related to ovary development and sexual maturation. However, studies on porcine ovarian genome-wide DNA methylation have not yet been conducted.

The four main sequencing technologies used for exploring genome-wide DNA methylation are the followings: methylated DNA binding domain sequencing^16^, methylated DNA immunoprecipitation sequencing^17^, whole genome bisulfite sequencing (WGBS)^18^ and reduced representation bisulfite sequencing (RRBS)^19^. The former two use the enrichment of methylated DNA to acquire a maximum resolution of 150 bp^20^, while the latter two achieve single-base resolution through the bisulfite conversion. Generally, methods using bisulfite conversion are more accurate than those using enrichment^20^,^21^. Compared with WGBS, RRBS is a cost-effective channel for studying genome-wide patterns of DNA methylation^20^,^21^. *Msp*I (C|CGG), a CpG motif-specific restriction enzyme, is used in RRBS to digest genomic DNA^22^,^23^ systematically to ensure that each fragment obtained contains at least one CpG site. Thereafter, the RRBS library prepared from the target fragments is sequenced to obtain the reduced representation methylome^24^,^25^. RRBS prefers CpG-rich regions, including CpG islands, promoters and enhancers^26^. Gradually, RRBS has been widely implemented for DNA methylome research in humans^27^ and model organisms^28^,^29^,^30^, but seldom in pigs.

Here, we aimed to perform RRBS on porcine ovaries and profile their genome-wide DNA methylation to analyze their methylome. First, a reduced representation (RR) genome was built based on our analysis of the porcine ovarian genome digested by *Msp*I *in silico*. After sequencing the RR genome (n = 3) by RRBS operating standards, we surveyed variations in the CpG sites (CpGs) captured from the RR genome. Second, a detailed DNA methylation profile was depicted from screening the methylation level of the porcine ovary and the methylation features of transcriptional start sites (TSSs), transcriptional end site (TESs), low CpG content promoter (LCP) genes, high CpG content promoter (HCP) genes and CpG islands (CGIs). Then the ovarian RRBS data from the present study were compared with the porcine intestinal RRBS data from one published study^31^. Our findings would advance the knowledge and understanding of the porcine methylome.

## Results

### The porcine RR genome

The porcine genome (Sscrofa 10.2) was digested by *Msp*I *in silico* to identify the appropriate fragment sizes for the RR genome. The total genome size was 2.8 Gb, and it contained 60.92 million CpGs in two strands (Table 1). We also downloaded human GRCh38 genome and mouse GRCm38 genome, and found 58.74 million CpGs in humans and 43.81 million CpGs in mice on two strands. There were 21,691.39 CpGs per Mb in the porcine genome (Table 1) which was higher than that in the human and mouse genome. For the porcine genome, it contained 2.37 million *Msp*I digested DNA segments. The segments per Mb were 842.77 (Table 1), and distributed asymmetrically among each of the porcine chromosomes (see Supplementary Figure S1a and Supplementary Table S1). The chromosome designated 0 in Supplementary Figure S1a represents the segments from scaffolds that could not be aligned to the porcine reference genome. The segment lengths had a peak density of 74 bp (see Supplementary Figure S1b).

**Figure 1:**
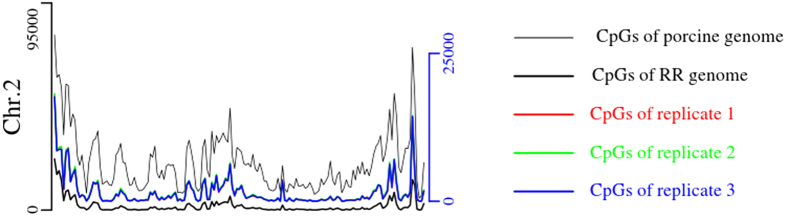
Distribution of CpGs on chromosome 2 (Chr.2). The thin black line represents the total distribution of the porcine CpGs on Chr.2. The thick black line represents the distribution of the theoretical CpGs on Chr.2 of the RR genome and shares the same vertical axis as the porcine genome, which stands on the left. Red, green and blue lines represents the distribution of the CpGs detected with the coverage of at least five reads on Chr.2 for the three ovarian replicates; these replicates share the vertical axis on the right. The CpG distribution in the three replicates was highly overlapping. The coverage of CpGs was counted by 1 Mb windows.

Based on our simulations, segments of 110–220 bp were used to build the RR genome. The distribution patterns of the digested segments and CpGs in the RR genome were compared with those in the porcine genome to search for correlations between them. The RR genome consisted of 0.28 million digested segments and was 45.25 Mb in length (Table 1). The number of digested segments per Mb was 6214.72 in the RR genome (Table 1), a 7.37-fold enrichment of the porcine genome. The Pearson’s correlation coefficient of the digested segments located at each chromosome between the RR genome and the porcine genome was 0.96 (*P* = 5.69 × 10^−12^), which implied that the RR genome shared a high correlation with the porcine genome (see Supplementary Table S1). Next, we profiled the CpG distribution per Mb for each chromosome in the porcine and RR genomes (see Supplementary Figure S2). The CpG distribution for chromosome 2 (Chr.2) was shown in Fig. 1. The CpG trend in the RR genome was highly coordinated with that of the porcine genome. Pearson’s correlation coefficient was 0.76 (*P* = 2.20 × 10^−16^). These results suggested that CpGs covered in the RR genome were a representative sample of all porcine CpGs with respect to gross chromosomal location.

Given that *Msp*I prefers CpG sites, we counted the CpGs in the porcine and RR genomes to investigate whether there was a CpG preference in the RR genome. There were 21,691.39 CpGs per Mb in the porcine genome and 82,086.76 per Mb in the RR genome (Table 1), suggesting that the RR genome had a 3.78-fold enrichment over that of the porcine genome.

Based on the gene location, the porcine genome was divided into five genomic features, including 5 kb regions upstream from the TSS (up5kb), exons, introns, 2 kb regions downstream from the TES (down2kb) and the intergenic regions (Table 1). 3.64% of CpGs in the porcine genome were located on chromosome 0. The RR genome contained 6.10% of the CpGs in the porcine genome (Table 1). These CpGs represented 10.58% of up5kb, 16.41% of the exons, 6.44% of the introns, 9.70% of down2kb and 5.29% of the intergenic regions. The number of CpGs differed among these five regions (Table 1).

### Sequencing, quality control and matching

The genomic DNA extracted from the ovaries of three Landrace × Yorkshire crossbred gilts aged 6 months were digested by *Msp*I, and the 110–220 bp digested fragments were selected to build the libraries. Then, the libraries were sequenced through the paired-end of 100 bp (PE100) on an Illumina HiSeq 2500 platform using RRBS operating standards^23^,^26^. Each sample generated approximately 42.40 million paired reads (see Supplementary Table S2). After quality control of the raw data, including removing adaptor pollution, multiple N sequences and low-quality sequences, each library produced approximately 7 Gb of clean data for further analysis (see Supplementary Table S2). The clean reads were aligned to the porcine genome by Bismark^32^. Reads aligned uniquely were used for further analysis. The aligned patterns of the unaligned, unique aligned and multiple aligned reads were in good agreement among the three libraries (see Supplementary Figure S3). The reads mapping to unique locations were 61.10%, 60.70% and 59.20% (see Supplementary Figure S3).

### Description of the captured CpGs

**Figure 2:**
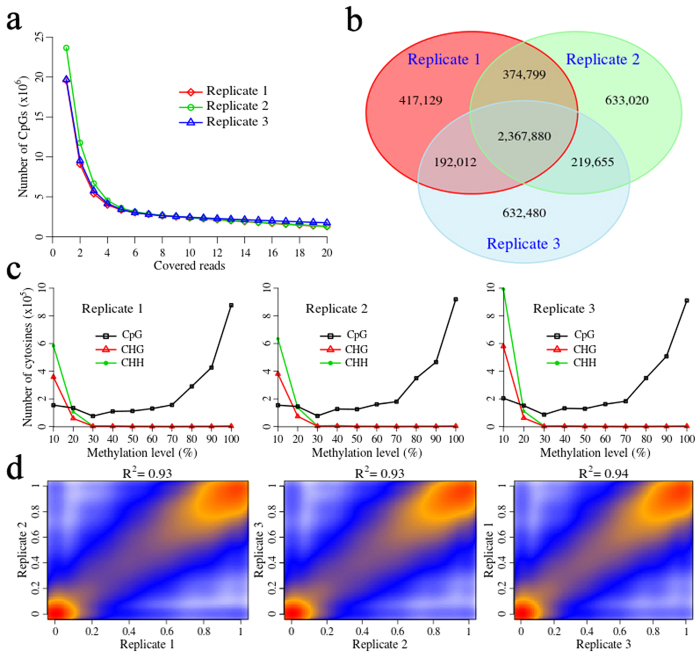
CpG site distribution in the three ovarian replicates. **(a)** Distribution of CpG sites at different read coverage. CpGs with a read coverage of more than five were retained for further analysis. **(b)** Number of CpGs matched within the three replicates. **(c)** Distribution of the number of CpGs, CHHs and CHGs at different methylation levels (H = C, T and A). **(d)** Pearson’s correlation coefficients of the methylation levels of the three replicates.

RRBS variations were surveyed in the three biological porcine ovarian replicates by screening the distribution and methylation patterns of the CpGs detected. First, the number of CpG sites were counted for different read depths, as shown in Fig. 2a. Approximately, each sample generated 21 million CpGs. The number of CpGs with five covered reads was similar among the three ovarian samples. We retained the CpGs with at least five covered reads for further analysis.

The CpGs with at least five covered reads were matched among the three ovarian replicates, and 2,367,880 CpGs were concurrent in three replicates (Fig. 2b). CpGs, CHHs and CHGs (H = C, T and A) were counted at different methylated levels (Fig. 2c). The methylation levels of CpGs had a bimodal distribution (Fig. 2c). Furthermore, most of the CpGs were 70–100% methylated, and the vast majority of cytosines in the CHG and CHH contexts were 10–30% methylated (Fig. 2c). The methylation patterns in the three ovarian replicates were similar to other species such as humans^33^ and mice^3^. Among the three biological replicates, the Pearson’s correlation coefficient of each CpG methylation level was 0.93 (*P* = 2.2 × 10^−16^), 0.93 (*P* = 2.2 × 10^−16^) and 0.94 (*P* = 2.2 × 10^−16^) (Fig. 2d). These results suggested that biological variation among the three ovarian replicates was extremely low. The methylation statuses of the three ovarian replicates from the three pigs were equally similar.

### RRBS reproducibility in the three ovarian replicates

**Figure 3:**
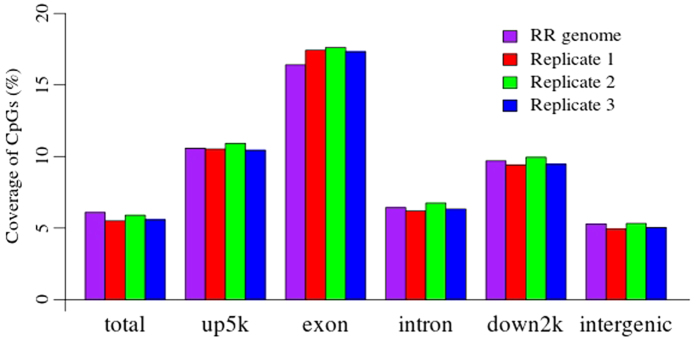
CpG coverage in the three ovarian replicates. Only the detected CpGs with at least a five-read coverage in the three ovarian replicates were used.

The reproducibility of RRBS was investigated by depicting the distribution of CpGs that were detected in the genome (see Supplementary Figure S2) and genomic features (Fig. 3) of the three ovarian replicates. The detected CpGs per Mb overlapped completely among three ovarian replicates (see Supplementary Figure S2). The profile of detected CpGs in Chr.2 was shown in Fig. 1. Pearson’s correlation coefficients of the trends in CpGs between the replicates and the porcine genome were 0.89 (*P* = 2.2 × 10^−16^), 0.90 (*P* = 2.2 × 10^−16^) and 0.90 (P = 2.2 × 10^−16^). Pearson’s correlation coefficients of the trends in CpGs between the replicates and the RR genome were all 0.77 (*P* = 2.2 × 10^−16^). Therefore, the trends of the CpGs in the replicates were almost identical to those of the porcine and RR genomes, indicating that RRBS was able to represent the genome-scale DNA methylation.

The CpGs detected by RRBS in the three ovarian replicates represented 5.50%, 5.89% and 5.59% of the total CpGs in the porcine genome, coming close to the theoretical magnitude of the RR genome (Fig. 3). Additionally, the CpGs for the five genomic features detected in the three replicates were almost the same as their theoretical value (Fig. 3).

### Methylation level of the porcine ovary genome

**Figure 4:**
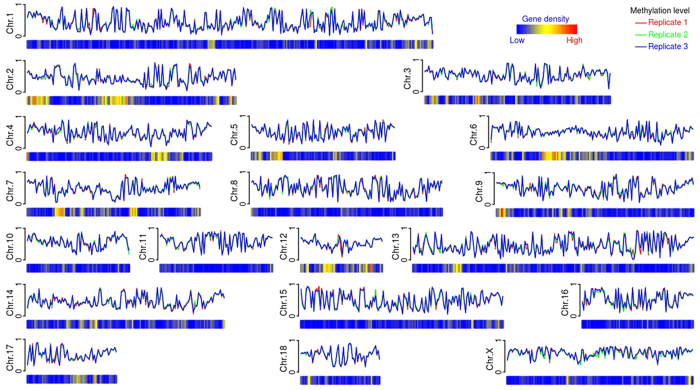
Methylation level of the porcine ovary genome. The methylation level and gene density were counted by 1 Mb windows.

The methylation level in the porcine ovary versus gene density was shown in Fig. 4. The methylation levels varied across the different chromosomes and regions. Hypermethylation was apparent in regions of low gene abundance, whereas hypomethylation was generally in those of high gene abundance. This suggested that genome hypomethylation was beneficial for promoting gene transcription. The methylation levels of the three ovarian replicates showed the same trends and overlapped greatly (Fig. 4), suggesting that the variation between biological replicates was low.

### CpG content and methylation around transcription start sites

**Figure 5:**
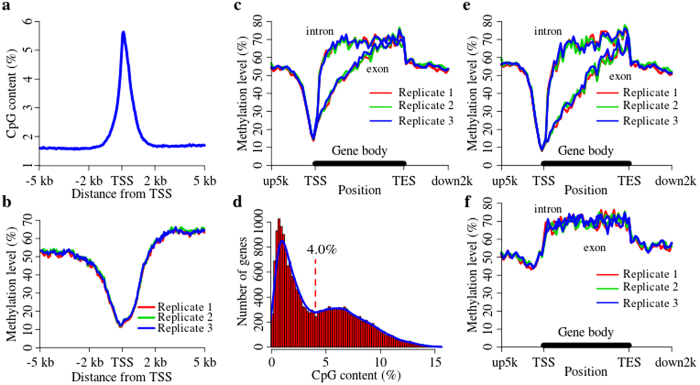
Methylation patterns of porcine genomic features. **(a)** CpG content around TSSs in the porcine genome. **(b)** Methylation levels of CpGs around TSSs in the porcine genome. **(c)** Methylation levels of whole genic features. **(d)** Distribution of the number of genes counted by CpG content in the 1000-bp region around TSSs (from −500 bp to +500 bp). The HCP and LCP gene cutoff was 4.0%. **(e)** Methylation levels of HCP genic features. **(f)** Methylation levels of LCP genic features.

The region around the TSS is crucial for regulating gene expression. We calculated the CpG content and methylation level of 5 kb upstream (−5 kb) and 5 kb downstream (+5 kb) around TSSs from all the protein-coding genes annotated in the porcine genome using 50 bp-length bins (Fig. 5a,b). The CpG content was highest at TSSs, thereafter decreasing sharply within 2 kb and plateauing steadily beyond 2 kb around TSSs (Fig. 5a). Furthermore, there was a negative correlation between the methylation levels around TSSs and the CpG content (Fig. 5b). The methylation at −5 kb was relatively higher until −2 kb, whereupon it decreased quickly and reached the lowest level at the TSSs. Methylation then increased after the TSSs, but fluctuated from +200 bp to +700 bp (Fig. 5b), in which the 5′UTR, the first exon or first intron were located.

### Methylation level at gene locations

The methylation levels of various genomic features, including up5kb, intron, exon and down2kb, were calculated to investigate the methylation patterns in locations containing whole genes (Fig. 5c). As shown in Fig. 5c, the methylation level was lowest at TSSs, but increased within the genes, especially in the front half of gene bodies. Moreover, it declined abruptly at the transformation point from the gene body to the region downstream. Furthermore, methylation levels were higher in introns than in exons, particularly in the first half of the gene body where the methylation level increased sharply. This finding was consistent with a previous study that the strong DNA methylation at promoters or the first exon of genes was associated with transcriptional silencing^34^, and the function of a higher methylation level within the gene body possibly did not block transcription but instead stimulated it^34^,^35^.

### Methylation of LCP and HCP genes

Promoters trigger gene transcriptions, but their DNA methylation status may result in transcriptional silencing^34^,^36^. The CpG content around TSSs (−500 bp to +500 bp) was calculated as the CpG content of the promoter (Fig. 5d). In common with that in humans^37^ and rats^29^, a bimodal distribution was also observed in pigs. Notably, the crossover point was 4.0% by fitting the two normal distributions; accordingly, we separated the porcine promoters into two classes: HCPs and LCPs. From the methylation patterns of the HCP genes shown in Fig. 5e, we found that the curve trends were similar to those of whole genes (Fig. 5c), but the methylation levels of the TSSs in the HCP genes were slightly lower in the all annotated genes. Moreover, the difference between the methylation level of introns and that of exons was not distinct (Fig. 5f) in the LCP genes. However, the methylation of LCP genes was essentially different from that of the HCP genes, especially for the methylation around TSSs.

### Methylation of CGIs

**Figure 6:**
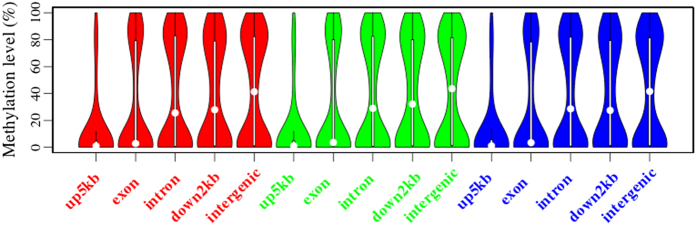
Methylation of CGIs located in relation to different genomic features. Red, green and blue represent the ovarian replicate 1, the ovarian replicate 2 and the ovarian replicate 3, separately.

CpG sites play an important function in regulating gene expression and mammalian evolution^37^,^38^. Generally, one CpG site arises every 100 bp in mammalian genomes. However, there are some regions where one CpG site arises every 10 bp; these regions are called CpG islands (CGIs)^39^,^40^. Evidences have indicated that CGIs encompass the TSSs of ~60% of human protein-coding genes and ~40% of tissue-specific genes^41^,^42^. Methylation of CpG islands located in promoters is associated with transcriptional silencing^34^. Additionally, CGIs in the body of genes can act as alternative promoters or enhancers and display tissue specific methylation^43^,^44^. The distribution and methylation patterns of CGIs in genomic elements were shown in Fig. 6. Most CGIs were located in the intergenic regions, and CGI methylation in up5kb was lowest. The CGI methylation in exons was obviously lower than that of CGIs in introns, down2kb and intergenic regions. Furthermore, the CGI methylation pattern was consistent with that of the location of whole genes. The CGI methylation patterns might contribute to cis-regulatory functions.

### Differential methylated patterns between ovary and intestine

**Figure 7:**
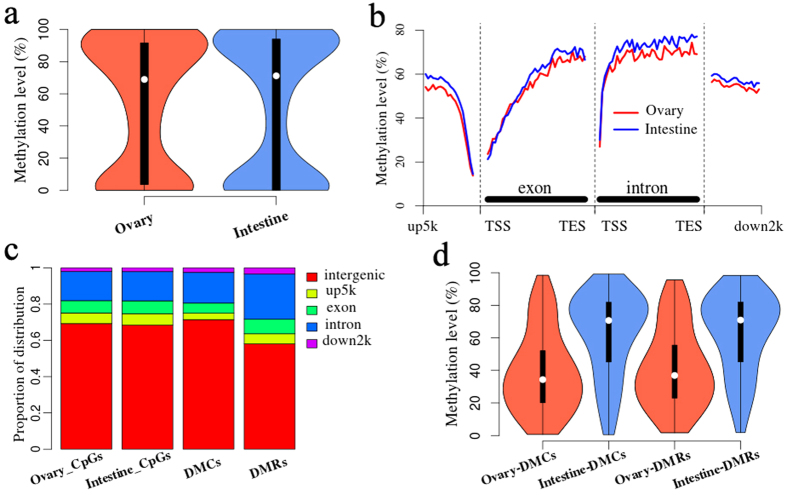
Differential methylated patterns between ovary and intestine. (**a**) Vioplot of the ovarian and intestinal methylation level. (**b**) Differential methylation levels between ovary and intestine of whole genic features. (**c**) Distribution of CpGs, differently methylated CpGs (DMCs), and differently methylated regions (DMRs). (**d**) Vioplot of DMCs and DMRs methylation levels in ovary and intestine.

The RRBS data of ovarian replicate 1 were compared with the porcine RRBS data of the full-term newborn intestine from one previous study^31^ (downloaded from GEO DataSets, 0d-term, GSM1299966) to identity putative porcine tissue specific DNA methylated patterns. The CpGs with more than five covered reads were used to screen the differential methylation between ovary and intestine. The average methylation level of the ovary was almost the same as that of the intestine (Fig. 7a). The methylated trend of gene locations around the TSS in ovary was highly concordant with that in intestine (Fig. 7b). The differential methylation disappeared gradually around the TSS, and then it enlarged along with the exons and introns as well as the down2k region (Fig. 7b).

Next, we carried out the R package “DSS”^45^ to identify the differently methylated CpGs (DMCs) and differently methylated regions (DMRs) between ovary and intestine. Totally, we identified 208,640 DMCs and 4,131 DMRs whose methylation levels varied more than 20% and the P value from the Wald test corrected by the false discovery rate was smaller than 0.01. We found that the mean length of DMRs was 360 bp and the mean number of CpGs covered in DMRs was about 30. The DMCs predominantly occurred in intergenic (71.40%), intronic (16.91%), and exonic (5.58%) regions (Fig. 7c). Compared with the distribution of covered CpGs in the ovary and the intestine, the proportion of DMCs located in up5k and exon decreased (Fig. 7c). In addition, the DMRs also occurred predominantly in intergenic (58.10%), intronic (24.91%), and exonic (8.03%) regions (Fig. 7c). Furthermore, the methylation levels of DMCs and DMRs in the ovary were lower than that in the intestine (Fig. 7d). Compared with intestine, there were 153,269 DMCs and 2,987 DMRs with less DNA methylation in ovary, and 55,371 DMCs and 1,144 DMRs with more DNA methylation.

## Discussion

The ovary plays a vital role in mammalian animal reproductive processes. Studies have shown that DNA methylation regulates sexual and ovarian maturation^12^,^13^. By profiling the methylome of the porcine ovaries, researchers can examine the methylation changes during the process of sexual and ovarian maturation in pigs. From the approaches available for acquiring genome-wide profiles of DNA methylation, RRBS and WGBS, which both use bisulfite conversion to generate single nucleotide resolution, are more attractive than enrichment-based methods. However, the cost effectiveness of WGBS is lower than that of RRBS^20^,^21^. For example, with a read depth of 28.2× and 29.6× for the human genome, WGBS yielded 87.5 and 91.0 Gb sequences, respectively^18^, while RRBS just required 5 Gb sequences for human genome^24^.

In mammals, methylated cytosines predominantly occur in CpG dinucleotides^46^. Because of the extensive non-CpG regions in the mammalian genome, the vast amount of data obtained by WGBS would be cost prohibitive. In sheep, for the ~2.5 Gb of data obtained by RRBS^47^, more than 1.8 million CpGs were detected and 600,000 of these had a depth of at least 10 × depth. However, with this sequencing depth, 400,000 CpGs were detected by WGBS and only 1065 of these were with >10 × depth^47^. Here, we built the porcine RR genome, with fragment sizes of 110–220 bp, to reduce the amount of sequencing required. The digested segments and CpG distribution in the RR genome had a high correlation with the porcine genome. Therefore, sequencing the porcine RR genome was cost-effective and represented the porcine genome-wide methylation profile well. Particularly, the CpGs captured by RRBS varied across the five genomic regions tested in the three ovarian replicates, but came close to the theoretical magnitude of the RR genome.

Studies on genome-wide methylation patterns by RRBS have been conducted in humans^27^, model organisms^28^,^29^,^30^ and sheep^47^. Notably, in these studies, the sequencing strategy determined the mapping efficiency. For instance, when RRBS is used with fragment sizes of 50–150 bp and read lengths of 100 bp, a mapping efficiency of 38.3% was found in sheep^47^. However, fragment sizes of 150–250 bp increased the mapping efficiency to 61.4%^47^. With this information, we simulated the different sequencing strategies on different size selections for RRBS and found that the promoter and CGI region coverage were best with a fragment size of 110–220 bp by PE100 (see Supplementary Table S3). Next, we performed RRBS by PE100 with 110–220 bp sizes on three ovarian replicates from pigs. The mapping efficiencies were about 60% for each replicates (see Supplementary Figure S3). Accordingly, the mapping was highly effective when sequencing the porcine RR genome by PE100.

RRBS have been applied on mammalian libraries of 40–220 bp^5^,^28^,^30^. Indeed, selection of a single sequencing strategy for 40–220 bp libraries is considered to be not sensible because the information obtained from the numerous fragments longer than 100 bp will drop off, with fragments of 40–220 bp and read lengths of 50 bp. Alternatively, sequencing fragment sizes of 40–220 bp with read lengths of 100 bp results in the pollution of a large amount of the data by the sequencing adapters.

The methylation patterns of porcine genes are analogous to those observed in other vertebrate animals such as humans^18^, bovines^48^, horses^49^ and chickens^50^. The methylated patterns were such that the methylation level of the gene body was higher than in its 5′ and 3′ flanking regions, with an increase from the TSS to TES, and there was a sharp reduction after the TES (Fig. 5c). The methylation level fluctuated at 200–700 bp after the TSS (Fig. 5b) where the 5′UTR, the first exon or first intron was located. These genomic regions had a large number of trans-acting factor binding sites. Methylated sites inhibit trans-acting factor binding^51^,^52^, and then regulate gene transcription. Methylation level in introns was heavier than that in exons (Fig. 5c), especially for the front half of a gene body. This might be the cause of the fluctuation observed at 200–700 bp (Fig. 5b). In one study, the CpG content of introns was lower than that of exons^37^, which might be the primary cause of the heavier methylation status of introns compared with exons. Moreover, this methylation difference might regulate pre-mRNA splicing by affecting the enrichment of CTCF^53^ and MeCP2^54^ on alternative splicing.

Each species has a species-specific genome, especially in terms of its promoters^37^. The frequency distribution of CpG content around TSSs had a bimodal distribution in pigs (Fig. 5d) and also in many other mammalian species such as rats^29^ and humans^37^. However, in some mammals such as sheep^55^ and cows^55^, there is no such bimodal distribution. Previous studies have shown that mammalian promoters are separated into two classes according to their CpG content, namely HCPs and LCPs^37^,^56^. Other studies have set three grades of promoters by CpG_o/e_ ratio, including low, intermediate and high CpG promoters^3^,^57^. The two classes of promoter patterns evolved in the earlier vertebrate evolution^37^,^56^. In pigs, the methylation of LCP genes was essentially different from that of the HCP genes (Fig. 5f), especially for the methylation difference around the TSSs and the difference between their own introns and exons. The methylated patterns of porcine HCP and LCP genes were similar to those around TSSs of ovine HCP and LCP genes^55^, respectively. Furthermore, the methylated patterns around TSSs of HCP and LCP genes in pigs were also similar to that of highly expressed HCP genes and repressed LCP genes in rats^29^, respectively. HCPs are strongly associated with housekeeping genes^28^,^57^ and are often hypomethylated, with methylation occurring by maintenance mechanisms^58^. In contrast, LCPs are strongly associated with tissue-specific genes^28^,^57^ and are generally hypermethylated with methylation occurring by the *de novo* mechanism^58^.

## Methods

### Sample preparation and ethics statement

Ovarian samples were collected from three 6-month-old female Landrace × Yorkshire crossbred gilts. Animal care and the experiments were conducted according to the Regulations for the Administration of Affairs Concerning Experimental Animals (Ministry of Science and Technology, China, revised in June 2004) and approved by the Animal Care and Use Committee of the South China Agricultural University, Guangzhou, China (approval number: SCAU#2013-10). The animals were reared in the same environment and were fed the same diet *ad libitum*. After being humanely killed, ovarian samples were frozen quickly in liquid nitrogen, and then stored at −80 °C. A quarter of an ovary from each pig was ground in a mortar containing liquid nitrogen. Thereafter, DNA was extracted from the thoroughly mixed powder using a DNeasy Blood& Tissue Kit (Qiagen, Beijing) according to the manufacturer’s instructions.

### Simulation of RRBS for the porcine genome

The porcine genome used herein was Sscrofa 10.2 acquired from Ensembl (http://asia.ensembl.org/Sus_scrofa/Info/Index). The cutting site of *Msp*I restriction enzyme was C|CGG. We captured segments between two consecutive restriction sites (C|CGG) and aligned the segments to the porcine genome. PE100 was chose to generate the genome wide methylation of porcine ovary. The effective read length of PE100 was 100–200 bp. Fragment sizes of 40–110 bp, 110–220 bp and 220–350 bp were selected for comparison to assess the simulation performance. Based on the limitations of the practical operation of next-generation sequencing, the coverage of the promoter and CpG island regions were compared with different fragment sizes by the paired-end of 50 bp and PE100 in the simulations (see Supplementary Table S3). Based on the coverage of promoters and CpG islands, the fragment sizes of 110–220 bp were selected to build the RR genome (see Supplementary Table S3).

The simulations of the different fragment sizes were accomplished by unlocking the DNA double strands so that the front 50 bp or 100 bp sequence of one DNA strand was captured by pair end. Sequences captured in this way were aligned to the promoter regions and CpG island regions. Promoter regions (downloaded from Ensembl) were 2 kb upstream from TSSs. CGI regions (also downloaded from Ensembl) were described by Ensembl as regions >200 bp with a C and G percentage >0.5, and a ratio of the observed CpG/expected CpG was >0.6, and the expected CpG was calculated as (GC content/2)^2^.

### RRBS library preparation and sequencing

RRBS technical processes were based on previously published RRBS studies^22^,^23^ after checking on the quality of the DNA extracted. Briefly, the purified genomic DNA was digested overnight with *Msp*I (New England Biolabs, USA). The sticky ends produced by *Msp*I digestion were filled with CG nucleotides, and 3′ A overhangs were added. Methylated Illumina sequencing adapters with 3′ T overhangs were ligated to the digested DNA following the manufacturer’s protocols, and the products obtained were purified. For RR genome, the 110–220 bp insert sizes were converted by bisulfite using an EZ DNA Methylation Gold kit (Zymo Research, USA). The libraries were PCR amplified and each library was sequenced using one lane of an Illumina HiSeq 2500 and 100 bp paired-end reads. Quality control of the data was undertaken using FastQC software (Babraham Bioinformatics). The first two nucleotides were trimmed from all the second read sequences to blunt-end the *Msp*I site. All reads were trimmed using a Phred quality score of 20 as the minimum, removing the adaptor pollution reads and multiple N reads (where N > 10% of one read). The trimmed sequences were mapped to the porcine reference genome (Sscrofa 10.2) using the default parameters of Bismark software (Babraham Bioinformatics)^32^. The bisulfite conversion rates were calculated as the number of covered cytosines in non-CpG that were converted, divided by the total number of covered cytosines in non-CpG. The conversion efficiencies of three ovarian replicates were 99.63%, 99.64% and 99.60%. For CpG sites, reads from both strands were combined to calculate the methylation levels. Uniquely mapped reads were retained for further methylation level calculations. The RRBS data were submitted to European Nucleotide Archive (accession number: PRJEB12143).

### RRBS data analysis

The simulations and calculations in this study were accomplished by R script (R Core Team, 2013, Austria). Cytosine sites covered by at least five reads were retained for further analysis. The cytosine methylation level was calculated as the number of C bases (methylated reads) divided by the total number of C bases (methylated reads) and T bases (unmethylated reads) at the same position of each individual cytosine. Genome-wide DNA methylation analysis was conducted on all of the annotated protein-coding genes. The average methylation and CpG content around transcription start sites (from −5 kb to +5 kb) were counted by 50 bp-length bins. The average methylation across all annotated genes was integrated, including up5kb, intron, exon and down2kb. Introns were totally integrated by 5′UTRs, introns and 3′UTRs against the positions of all the annotated genes, following equal divisions into 40 bins. Similarly, exons were totally integrated against the exon positions for all the annotated genes, following equal division into 40 bins. Up5kb or down2kb were divided into 20 bins. The RRBS data of the full-term newborn porcine intestine (0d-term, GSM1299966) from one previous study^31^ were downloaded from GEO DataSets.

## Additional Information

**How to cite this article**: Yuan, X.-L. *et al*. Profiling the genome-wide DNA methylation pattern of porcine ovaries using reduced representation bisulfite sequencing. *Sci. Rep*. **6**, 22138; doi: 10.1038/srep22138 (2016).

## Supplementary Material

Supplementary Information

## Figures and Tables

**Table 1:** Digested segments and CpG sites in the porcine and RR genomes.

	Porcine genome	RR genome	Number of CpGs	Porcine genome	RR genome
Segment counts	2,366,953	281,216	Up5k	1,840,501	194,657
Genome size (Mb)	2,808.53	45.25	Exon	1,284,565	210,778
Number of segments/Mb	842.77	6214.72	Intron	8,554,267	550,909
Total number of CpGs	60,920,863	3,714,426	Down2k	732,923	71,086
Number of CpGs/Mb	21,691.39	82,086.76	Intergenic	46,292,140	2,450,550

The upstream 5 kb (up5k) was defined as the 5 kb region upstream of the transcription start site. Introns and exons were parsed from RefSeq annotation. The downstream 2 kb region (down2k) was 2 kb downstream of the transcription end site.
