# Supplementary material for: Profiling the genome-wide DNA methylation pattern of porcine ovaries using reduced representation bisulfite sequencing
Source: Sci Rep. 2016 Feb 25;6:22138. doi: 10.1038/srep22138 (PMC4766444; doi:10.1038/srep22138)
Supplement: Supplementary Information [file srep22138-s1.pdf]

Profiling the genome-wide DNA methylation pattern of porcine ovaries using reduced representation bisulfite sequencing

Xiao-Long Yuan[1+], Ning Gao[1+], Yan Xing[1], Hai-Bin Zhang[1], Ai-Ling Zhang[2], Jing Liu[1], Jin-Long He[1], Yuan Xu[1], Wen-Mian Lin[3], Zan-Mou Chen[1], Hao Zhang[1], Zhe Zhang[1*] & Jia-Qi Li[1*]

[1]Guangdong Provincial Key Lab of Agro-Animal Genomics and Molecular Breeding, National Engineering Research Centre for Breeding Swine Industry, College of Animal Science, South China Agricultural University, Guangzhou, Guangdong, China

[2]College of Biological and Food Engineering, Guangdong University of Education, Guangzhou, Guangdong, China

[3]Guangzhou Ribobio Co., Ltd., Guangzhou, Guangdong, China.

*Corresponding authors. Jia-Qi Li, E-mail: jqli@scau.edu.cn; Zhe Zhang, E-mail: zhezhang@scau.edu.cn.

[+]These authors contributed equally to this work.

Supplementary Table S1 | Distribution of the digested fragments in swine genome and RR genome

Chromosome	Length of Chromosome (Mb)	Swine genome		RR genome	
		Segment counts	Segment counts/Mb	Segment counts	segment counts/Mb
1	315.32	201,341	638.53	20,953	66.45
2	162.57	146,458	900.89	18,764	115.42
3	144.79	164,876	1138.73	22,735	157.02
4	143.47	118,906	828.79	13,157	91.71
5	111.51	99,471	892.04	11,863	106.39
6	157.77	191,672	1214.88	27,136	172.00
7	134.76	134,649	999.18	16,700	123.92
8	148.49	94,072	633.52	9,382	63.18
9	153.67	118,154	768.88	12,894	83.91
10	79.10	76,940	972.69	8,906	112.59
11	87.69	74,654	851.34	8,998	102.61
12	63.59	108,522	1706.59	16,646	261.77
13	218.64	121,400	555.25	10,782	49.31
14	153.85	139,814	908.77	16,115	104.74
15	157.68	95,042	602.75	9,497	60.23
16	86.90	57,391	660.43	5,953	68.50
17	69.70	74,034	1062.18	9,366	134.38

18	61.22	64,644	1055.93	8,185	133.70
X	144.29	98,805	684.77	10,342	71.68
Y	1.64	705	429.88	67	40.85
MT	0.02	18	900.00	1	50.00

Supplementary Table S2 | Quality control of raw data

Samples	Reads	Total reads	Removal of adaptor pollution (%)	Removal of multiple N sequences (%)	Removal of low quality sequences (%)	Q30(%)	Clean reads
**Sample 1**	Read-1	37,903,255	92.57%	92.55%	87.91%	84.13%	63,344,776
	Read-2	37,903,255	98.84%	98.77%	86.76%	90.30%	(80.79%)
**Sample 2**	Read-1	46,392,130	95.38%	95.33%	90.57%	89.86%	77,254,392
	Read-2	46,392,130	99.30%	99.29%	86.16%	84.08%	(81.42%)
**Sample 3**	Read-1	42,913,094	98.31%	98.31%	92.90%	89.12%	74,487,604
	Read-2	42,913,094	99.15%	99.08%	90.34%	87.64%	(86.79%)

Supplementary Table S3 | Coverage of different sequencing strategies on different fragment sizes in silicon

**Fragment size**	**40-110 bp**	**110-220 bp**	**220-350 bp**
**Read length**	**PE50**	**PE100**	**PE100**
**CpG island size of swine genome (Mb)**	**27.23**	**27.23**	**27.23**
Covered CpG island (Mb)	5.27	9.72	3.16

Coverage of CpG island (%)	19.35	35.70	11.61
**Promoter size of swine genome (Mb)**	**58.51**	**58.51**	**58.51**
Covered promoter region (Mb)	1.88	3.87	1.75
Coverage of promoter region (%)	3.21	6.61	2.99

Sequencing strategies were the pair end of 50 bp (PE50) and the pair end of 100 bp (PE100).

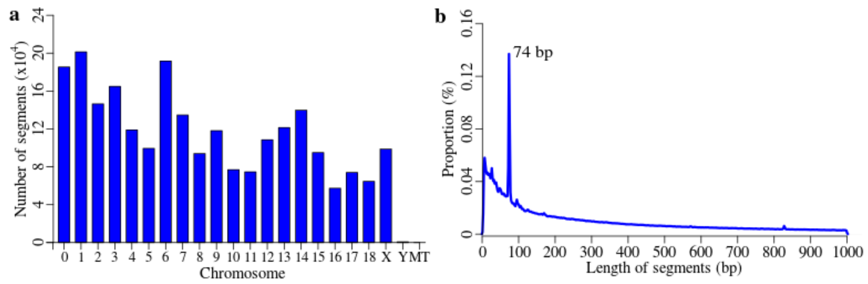

Supplementary Figure S1 | *Msp*I digestion of the porcine genome. **(a)** The number of segments digested by *Msp*I distributed on each chromosome. The chromosome designated 0 represented the segments from scaffolds that could not be aligned to the porcine reference genome. (**b**) The frequency distribution of segment length after digestion with *Msp*I.

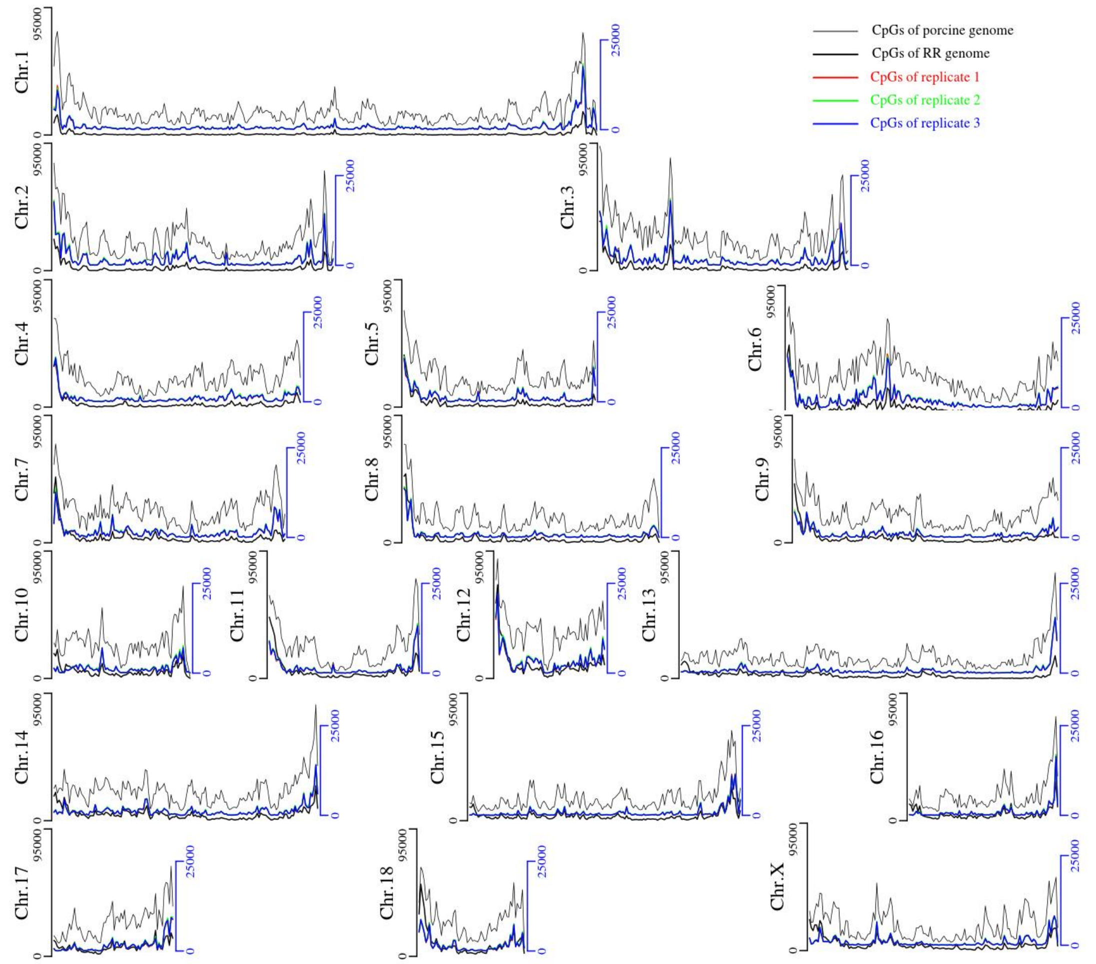

Supplementary Figure S2 | Distribution of CpGs on genome. The thin black line represented the total distribution of porcine CpGs on genome. The thick black line represented the distribution of theoretical CpGs on the RR genome and shared the same vertical axis as the porcine genome, which stood on the left. Red, green and blue lines represented the distribution of the CpGs detected with the coverage of at least five reads on genome for the three ovarian replicates; these replicates shared the vertical axis on the right. The CpG distribution in the three replicates was highly overlapping. The coverage of CpGs was counted by 1 Mb windows.

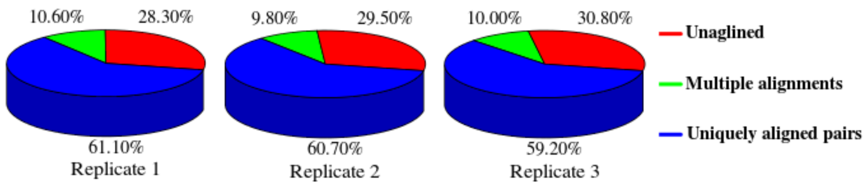

Supplementary Figure S3 | Concordance of the clean data.
